# Supplementary material for: All-at-once RNA folding with 3D motif prediction framed by evolutionary information
Source: Nat Methods. 2025 Oct 3;22(10):2094–106. doi: 10.1038/s41592-025-02833-w (PMC12510878; doi:10.1038/s41592-025-02833-w)
Supplement: Supplementary file 3 — Source code to run CaCoFold-R3D. Data and results. [file 41592_2025_2833_MOESM3_ESM.gz › supplemental_material/results/CaCoFold-R3D/CaCoFold-R3D_Rfam_vshuffle/Rfam.rscape.times.pdf]

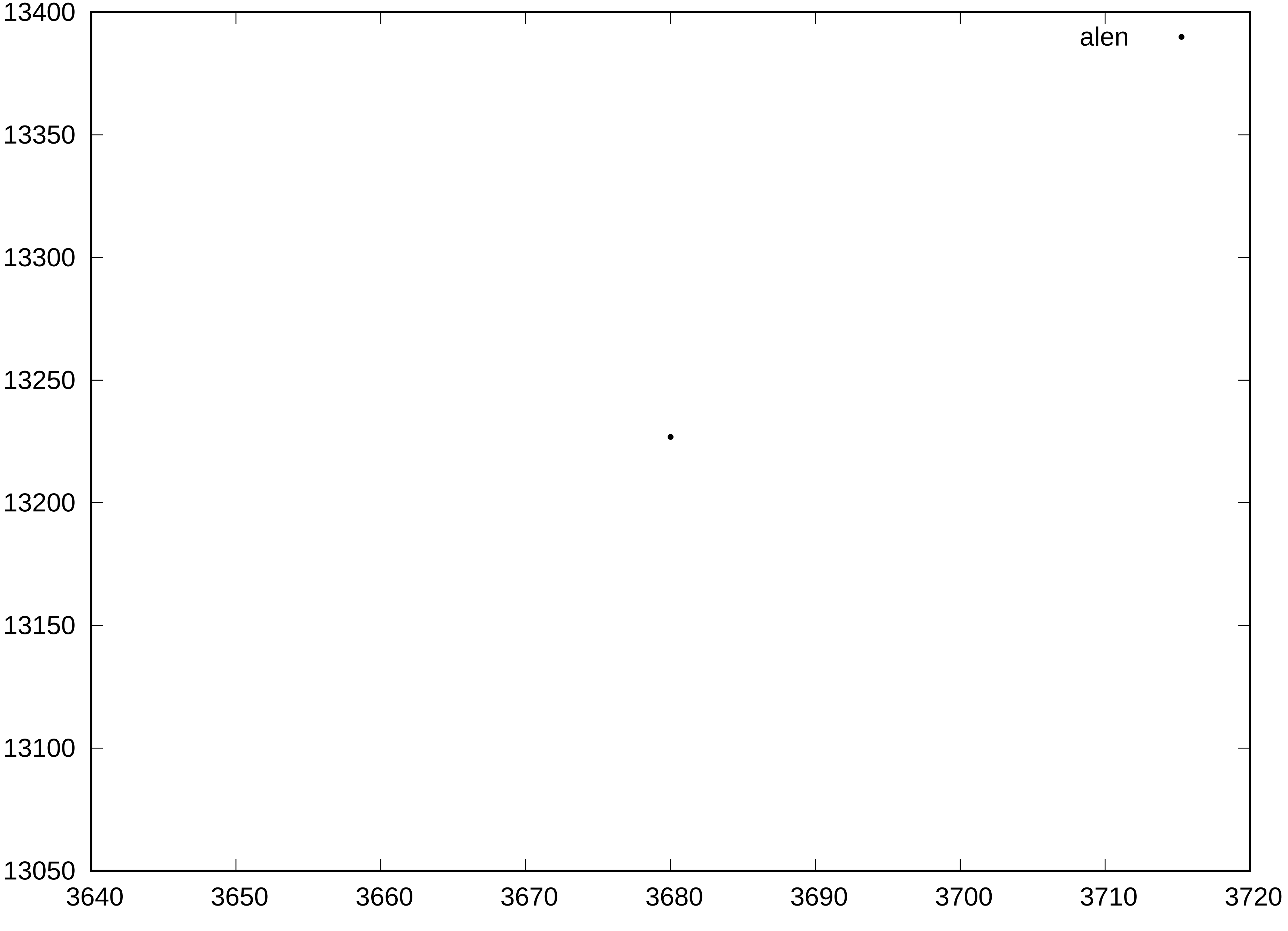

alen
13400
13350
13300
13250
13200
13150
13100
13050
3640
3650
3660
3670
3680
3690
3700
3710
3720

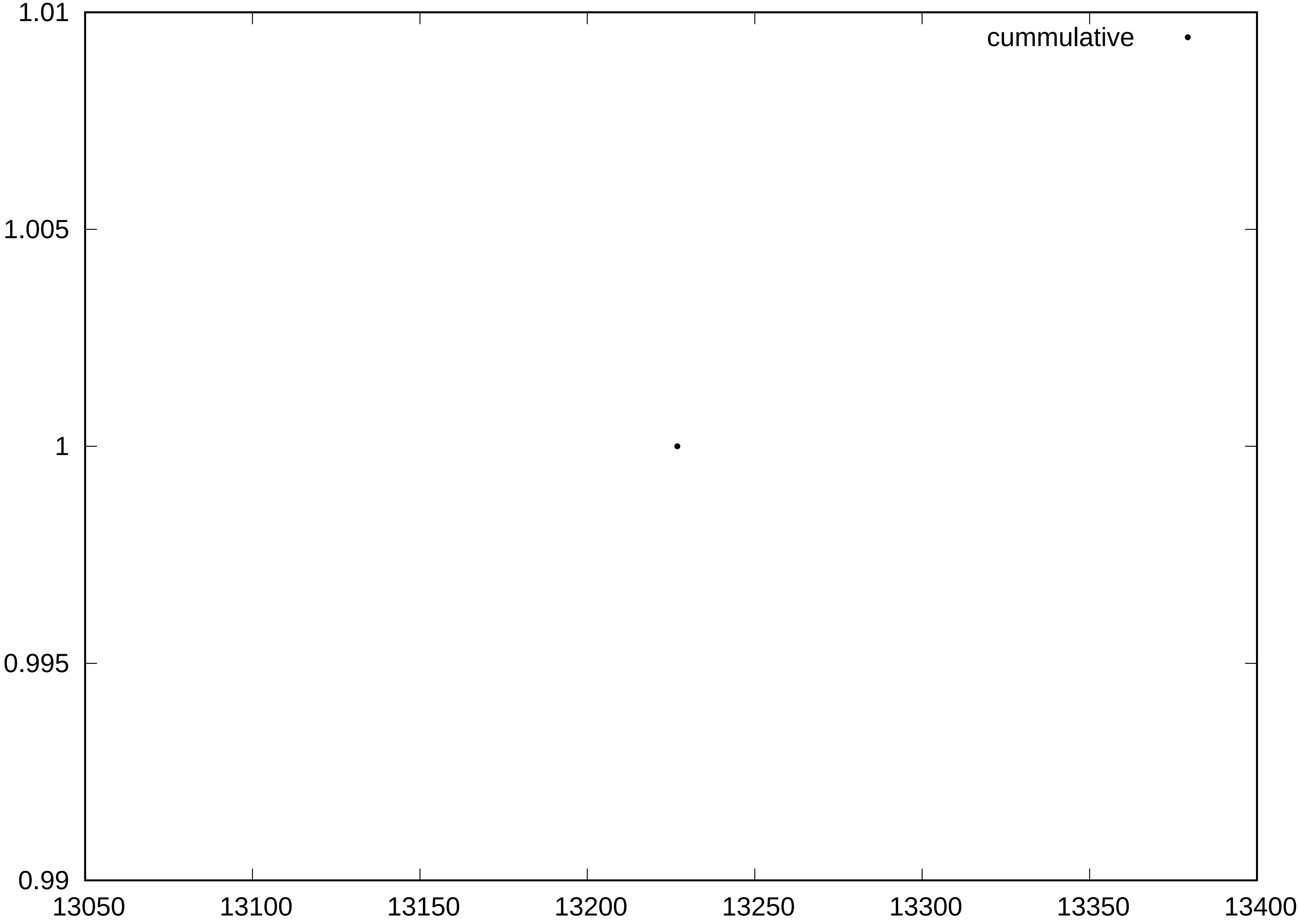

1.01
1.005
1
0.995
0.99
13050
13100
13150
13200
13250
13300
13350
13400
cummulative

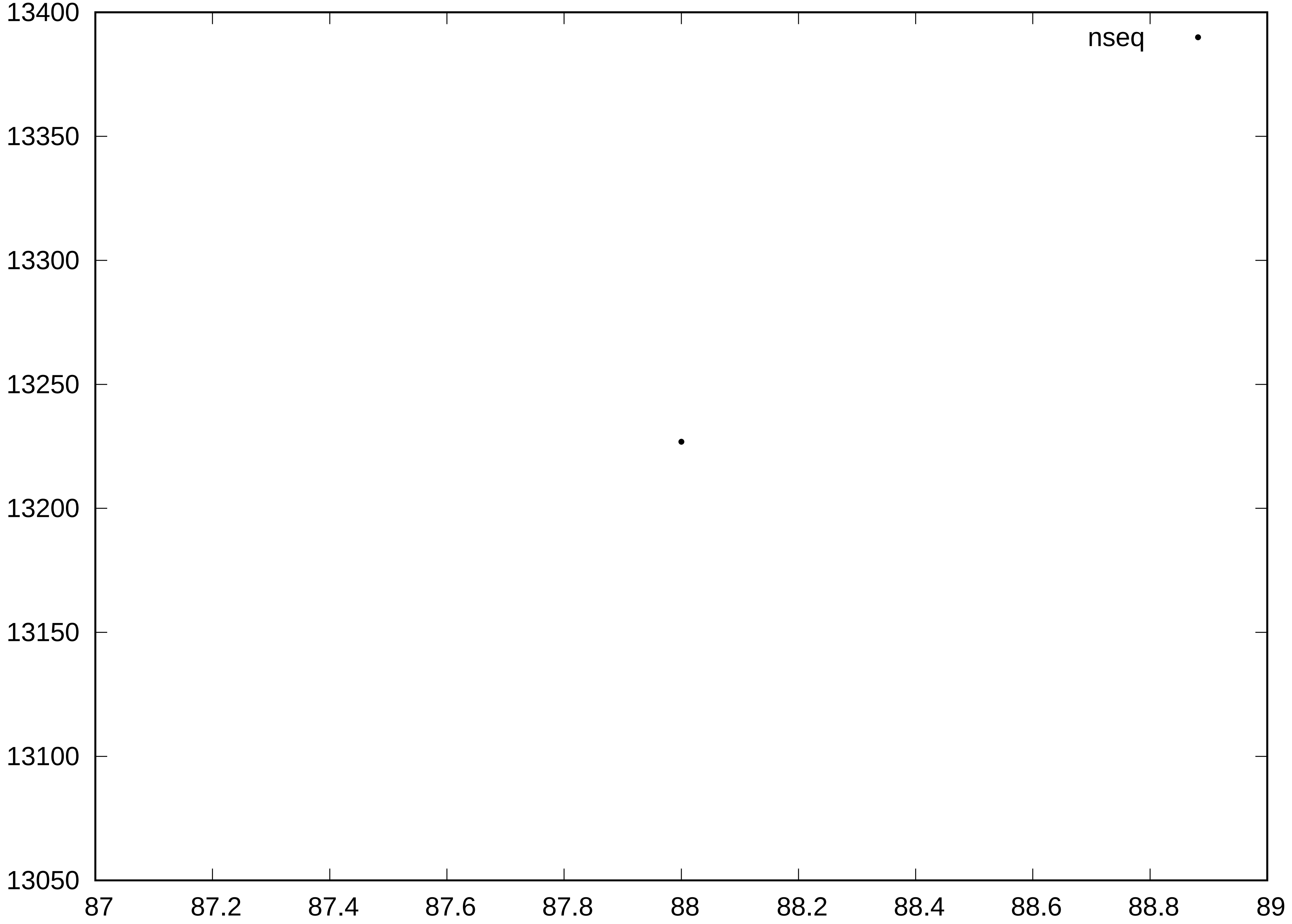

nseq
13400
13350
13300
13250
13200
13150
13100
13050
87
87.2
87.4
87.6
87.8
88
88.2
88.4
88.6
88.8
89

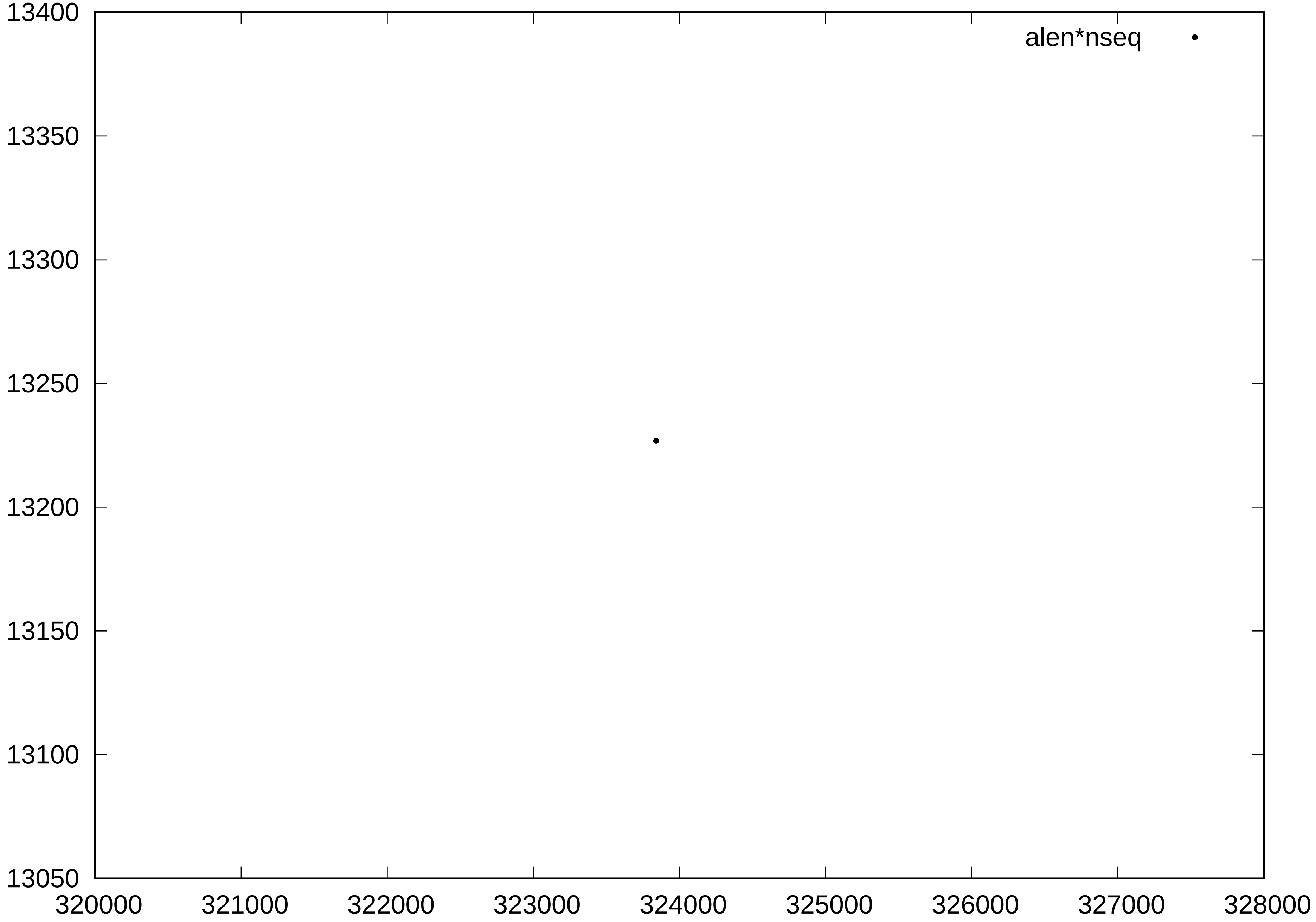

13400
13350
13300
13250
13200
13150
13100
13050
320000
321000
322000
323000
324000
325000
326000
327000
328000
alen*nseq
